# Sensory receptor repertoire in cyprid antennules of the barnacle *Balanus improvisus*

**DOI:** 10.1371/journal.pone.0216294

**Published:** 2019-05-02

**Authors:** Anna Abramova, Magnus Alm Rosenblad, Anders Blomberg, Tomas Axel Larsson

**Affiliations:** 1 Department of Chemistry and Molecular Biology, University of Gothenburg, Gothenburg, Sweden; 2 Department of Marine Sciences, University of Gothenburg, Gothenburg, Sweden; Monell Chemical Senses Center, UNITED STATES

## Abstract

Barnacle settlement involves sensing of a variety of exogenous cues. A pair of antennules is the main sensory organ that the cyprid larva uses to explore the surface. Antennules are equipped with a number of setae that have both chemo- and mechanosensing function. The current study explores the repertoire of sensory receptors in *Balanus improvisus* cyprid antennules with the goal to better understand sensory systems involved in the settling behavior of this species. We carried out transcriptome sequencing of dissected *B*. *improvisus* cyprid antennules. The generated transcriptome assembly was used to search for sensory receptors using HMM models. Among potential chemosensory genes, we identified the ionotropic receptors IR25a, IR8a and IR93a, and several divergent IR candidates to be expressed in the cyprid antennules. We found one gustatory-like receptor but no odorant receptors, chemosensory or odorant-binding proteins. Apart from chemosensory receptors, we also identified 13 potential mechanosensory genes represented by several transient receptor potential channels (TRP) subfamilies. Furthermore, we analyzed changes in expression profiles of IRs and TRPs during the *B*. *improvisus* settling process. Several of the sensory genes were differentially expressed during the course of larval settlement. This study gives expanded knowledge about the sensory systems present in barnacles, a taxonomic group for which only limited information about receptors is currently available. It furthermore serves as a starting point for more in depth studies of how sensory signaling affects settling behavior in barnacles with implications for preventing biofouling.

## Introduction

The barnacle *Balanus improvisus* Darwin 1854 *(= Amphibalanus improvisus)* is a common fouling species in temperate waters that, together with *Balanus amphitrite (= Amphibalanus amphitrite)*, has become a model organism for developing and testing antifouling coatings [1]. The barnacle life cycle involves sessile adults and planktonic larvae, including six naupliar larval stages followed by a cyprid larva that settles and metamorphoses into the sessile stage. Settling cyprids show a complex searching behavior that includes several distinct stages; e.g. free swimming, wide search, close search and inspection [2,3]. Surface exploration involves sensing of a variety of physical and chemical exogenous cues [4,5].

Adult barnacles have quite simplified sensory organs due to their sessile lifestyle. Apart from light-sensitive ocelli, the main sensory organs of adults are the thoracopods, called cirri, also used for suspension feeding. The cirri have distinct functionality depending on the various types of mechano-, thermo-, hygro- and chemosensory setae they carry [6]. In contrast to adults, cyprids that actively swim and explore the environment are equipped with more complex sensory organs such as a pair of compound eyes, frontal filaments, lattice organs, as well as the pair of antennules. It is believed that exploration of the substratum predominantly relies on the antennules. Cyprid antennules posses an attachment disc densely covered with cuticular villi and a number of setae that perform chemosensory and mechanosensory functions [7,8]. Some setae have a terminal pore and are suggested to perform contact chemoreception, while others are sac-shaped and thin-walled, called aesthetascs, that potentially can sense waterborne compounds [7,9]. It has been suggested that the majority of chemosensory setae, except the aesthetascs, are bimodal and also have a mechanoreceptive function [8]. However, the nature of the actual receptors involved in sensing settlement cues has remained elusive. Identification of barnacle sensory receptors would be a considerable asset and a step forward, both for studying external factors affecting the settlement behavior as well as for the development of new antifouling technologies.

Despite crustaceans being used as models to study olfaction, the repertoire of the chemosensory receptors remains largely unknown. In the majority of invertebrates studied in detail so far, chemoreception is most often mediated by three different gene families. These include two families of seven transmembrane receptors, namely the odorant receptors (ORs) and gustatory receptors (GRs), and a third family of ligand-gated ion channels, called the ionotropic receptors (IRs) [10]. Previous searches in arthropods revealed that ORs are only present in Hexapoda and seems to be lacking in all other groups [11–14]. GRs were suggested to be the most ancient family of chemosensory genes in arthropods, however currently only limited data are available for crustaceans with ten GRs found in *Eurytemora affinis* (Copepoda) [12], 58 in the *Daphnia* genome [14] and one candidate gene reported from the *B*. *amphitrite* transcriptome [12]. Initally discovered in *Drosophila* [15], IRs have thereafter been found in several Protostomes, suggesting that they are an ancestral chemosensory receptor family originating from the larger family of ionotropic glutamate receptors (iGluRs) [16]. IRs have a similar domain organisation to the iGluRs but are lacking one or more residues known to bind glutamate, suggesting that they have evolved to recognise other types of molecules. IRs act in complexes containing up to three subunits, including one or two common co-receptors, i.e. IR25a and IR8a, and individual odor-specific IRs [17]. Insect IRs are traditionally divided into two groups: “antennal” IRs, that were originally discovered in *Drosophila* antennal olfactory neurons, and species-specific “divergent” IRs, mainly expressed in the gustatory organs and involved in the detection of taste [16]. Importantly, earlier studies of antennal transcriptomes suggest that IRs are the only chemoreceptors found in crustacean antennas [10,11,18], therefore making IRs the potential candidate for detection of chemical cues during cyprid settlement.

Apart from chemical cues, it has been suggested that cyprids can distinguish surface structures by means of mechanosensory setae [8,9]. According to current knowledge, mechanoreception in arthropods is accomplished through mechanosensitive ion channels (transporting Ca^2+^ or Mg^2+^) that are gated upon mechanical stress and allow the influx of calcium ions resulting in a receptor potential [19]. Transient receptor potential (TRP) channels play major roles in various sensory modalities such as hearing, hygrosensation, vision and mechanosensation in a diverse set of animals [20]. Based on the amino acid sequences and the presence of specific domains, TRP channels have been divided into seven subfamilies—TRPA, TRPC, TRPM, TRPML, TRPN, TRPP and TRPV [20]. Among arthropods, TRPs have been mainly studied in insects and several of them were functionally characterised, e.g. *Drosophila* TRPN and TRPA have been shown to be involved in larval crawling behavior and thermosensation [20,21]. Several types of TRPs have been identified in *D*. *pulex*, however, their exact function in this species is unknown [20]. Based on behavioral studies and the use of chemical activators/inhibitors, it has been suggested that cyprid surface exploration rely on mechanosensitive Ca^2+^ channels, in particular involving the *D*. *melanogaster* homologs of *painless* and TRPA1 [22].

**Fig 1:**
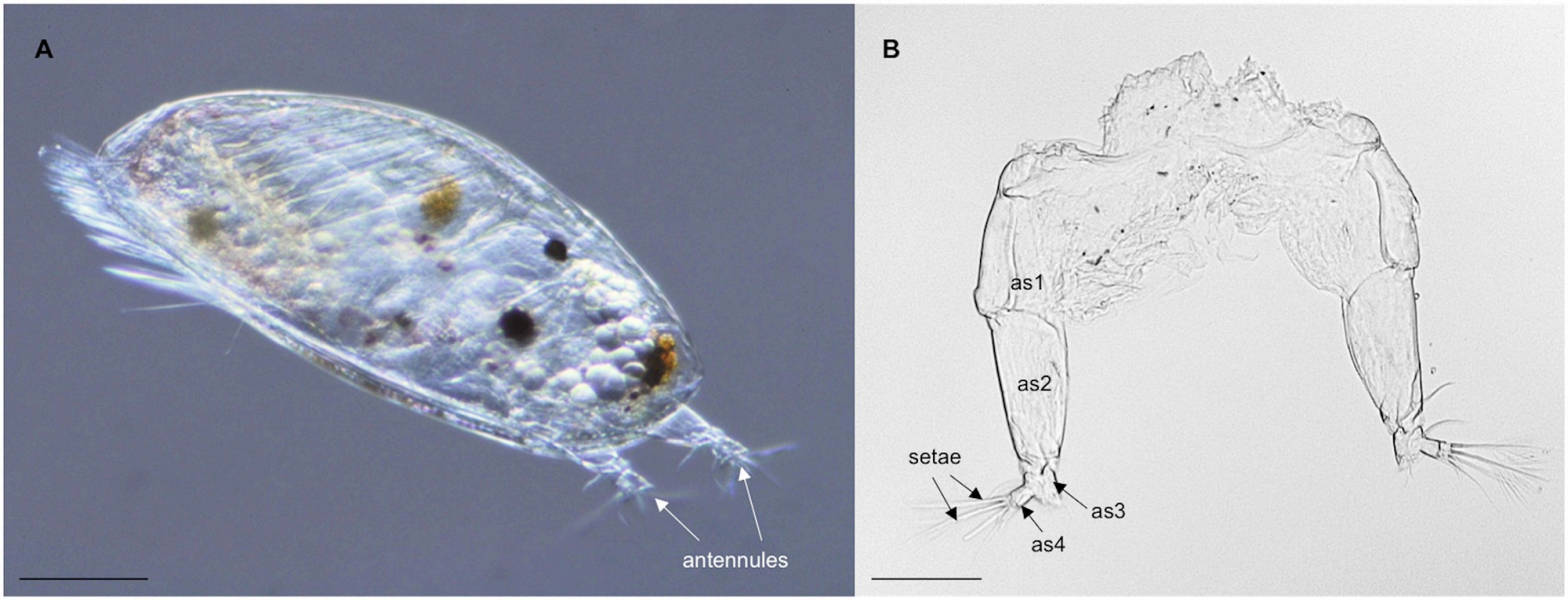
*B*. *improvisus* cyprid larva and a pair of antennules. (A) Lateral view of a cyprid with the pair of antennules indicated, scale bar = 100 μm. (B) a dissected pair of antennules with sensory setae and antennular segments (as1–4) indicated, scale bar = 50 μm.

No chemo- or mechanosensory receptors have been characterized in barnacles, except one partial GR-like gene found in *B*. *amphitrite* [12]. There are several studies showing involvement of serotonin and octopamine receptors in settlement and metamorphosis, however, it is not certain if these receptors are involved in the sensing of external settling cues [23,24]. Enrichment for chemosensory tissue followed by transcriptome sequencing proved to be a successful strategy to identify sensory receptor candidates [18,25,26]. We therefore generated transcriptome data from cyprid antennules in order to enrich for sensory genes (Fig 1). We made curated HMM models for sensory genes and performed bioinformatics searches in *B*. *improvisus* antennular dataset. Furthermore, we characterized the expression patterns of these sensory genes at four time points during the settlement process. As a result, we identified several differentially expressed genes encoding potential chemo- and mechanoreceptors. This study forms a basis for a better understanding of the barnacle sensing during settling and provides a foundation for development of more advanced antifouling technologies.

## Materials and methods

### Dissection of antennules and RNA extraction

Cyprid larvae were reared in an all-year-round laboratory culture of *B*. *improvisus* as described in Jonsson et al., 2018 [27]. Free swimming cyprid larvae, approximately 2–3 days old, were collected, placed in RNAlater (Ambion), and stored at 4°C for two days. Subsequently, individual larvae were placed on a glass slide and the pair of antennules was separated at the base of the proximal segment (see Fig 1) by means of stainless steel insect pins (Fine Science Tools, size 000, #26000–25) under a stereomicroscope (Bresser Researcher, WF 10X/20, 4X). Each pair of antennules was then transferred into the RLT buffer supplied with the RNeasy micro kit (Qiagen, GmbH, Hilden, Germany, #74004). In total, three independent samples were collected each containing antennular pairs from 50 individual cyprids. Samples were homogenized with Precellys Lysing Kit for hard tissue (CK28, Bertin Technologies, Montigny-le-Bretonneux, France, KT03961-1-002.2) and RNA extracted following the RNeasy micro kit (Qiagen, GmbH, Hilden, Germany, #74004) standard procedure.

### RNA-library preparation protocols and sequencing

The three independent RNA samples were pooled together into one sample to obtain enough RNA material for RNA library preparation (we obtained in total ≈ 1.2 μg RNA) and sent to the Science for Life Laboratory in Stockholm for sequencing. The cDNA Library was prepared according to the Illumina TruSeq Stranded mRNA Library Prep Kit (RS-122-2103) protocol with poly-A selection, and then sequenced on one lane of Illumina HiSeq 2500 to generate 260 million read pairs (126bp paired-end reads; in total roughly 89Gbp). The raw sequence data was uploaded to sequence read archive (SRA) with accession number SRR8755479.

### QC, assembly and Gene Ontology classification of sequencing data

The obtained antennular sequence data was initially quality checked using the software FastQC 0.8.0, Trim Galore! (http://www.bioinformatics.babraham.ac.uk/) and Cutadapt [28]. De novo transcriptome assembly was done using Trinity 2.0.6 [29] (with default settings) resulting in 239,215 contigs. The assembly was further clustered with cd-hit (v4.6.5) [30] with similarity threshold 0.95 to remove redundant transcripts resulting in 214,110 Trinity transcripts. The assembly was uploaded to the transcriptome shotgun assembly (TSA) database with BioProject ID PRJNA528169. We evaluated the completeness and duplication level of the assembly with arthropoda BUSCO v.2 [31], comprising 113 arthropod species and 1066 single copy orthologs. Transdecoder (v3.0.1) [32] was used to predict the coding regions in the transcripts, resulting in 15,931 complete ORFs and 35,284 internal (missing N- and C-terminal), while the rest were classified as 5’ and 3’ partials. GOSeq [33] inside the Trinity wrapper was applied to analyse gene ontology (GO) terms representation to score overrepresentation/underrepresentation of molecular function, biological process and cellular components. The GO terms were further passed to the web-based CateGOrizer (https://www.animalgenome.org/tools/catego/) software that batch analyses GO annotations by mapping them to the generic GO slim subset.

### Construction of HMMs

Searching with Hidden Markov Models (HMMs) is a more sensitive approach for identification of distant homologs than ordinary BLAST searches. We made separate HMMs for eight classes of the sensory receptors to be analysed, including both chemo- and mechanoreceptors. In particular, individual models were made for the different subtypes of ionotropic receptors (IR7a, IR8a, IR25a and IR93a) as they are the main candidates for olfactory receptors in crustaceans [11]. To search for classical chemosensory insect receptors we also made models for gustatory and odorant receptors, as well as for odorant-binding proteins (OBP), while for chemosensory proteins we used an HMM model downloaded from the PFAM database (PF03392).

HMM models were initially based on crustacean sequences previously reported as sensory receptors. Currently there are a limited number of sensory genes reliably annotated and functionally characterised in crustaceans. Because of this lack of information, we also included insect sensory receptors, and these are overrepresented in our final HMM models (see S1 Table for details). Alignments of sequences were performed with Mafft [34] and visualised with Jalview 2.10.1 [35]. Manually curated alignments for each receptor type were used to construct HMMs with HMMER v3.1b2 [36] to be used for searches against the predicted ORFs from the barnacle transcriptomes.

### Sensory gene candidates identification and the filtering process

For the HMM searches we used the antennular transcriptome dataset generated in the current study, as well as RNA-seq datasets previously generated from either free swimming cyprids or an adult of *B*. *improvisus* [37]. Firstly, from each of these datasets we extracted ORFs from the transcriptomes using Transdecoder v3.0.1. The created HMM models were used to mine the obtained ORFs for sensory receptor candidates. Given the shared domains between several of the included receptor families, one expects the HMMs to pick up some false positive hits. To filter out the false positives and retain the best predictions for a specific receptor class, we concatenated all the results sorted based on the E-value, and the hit with the lowest E-value from each HMM search was kept. The resulting top candidates were then compared against the NCBI nr database using BLASTP to further confirm the receptor identity and to exclude any false positives, i.e. making sure the best hit from the HMM search indeed belonged to the right receptor class annotation. Furthermore, we used the SMART (http://smart.embl-heidelberg.de) [38] domain search web-server to predict the domain content of the candidate receptor sequences from *B*. *improvisus*.

The initial searches in the *B*. *improvisus* antennule transcriptome revealed several short hits that despite the short length could be identified as potential sensory receptors. In an attempt to extend the sequence of these partial candidates, we used available Illumina and PacBio RNA datasets from the adults and cyprids of *B*. *improvisus* [37,39]. These extended candidates were further checked against genomic data (unpublished) to verify sequences. The sequences have been deposited in GenBank with accession numbers MK093193 –MK093208.

### Sequence and phylogenetic analysis of identified candidates

Identified candidate receptors were aligned with arthropod receptor sequences from NCBI nr database (see S2 File for the accession numbers). Phylogenetic trees based on the resulting alignments were made with PhyML method at phylogeny.fr (http://www.phylogeny.fr). The receptor candidates were named according to amino acid similarity to previously identified *Drosophila* and *Panulirus argus* IRs [11,15] as well as classification from the phylogenetic analysis results.

### Expression analysis of candidate genes during settlement

The candidate sensory receptors were analysed during a developmental time series experiment to identify their expression changes during settlement. The details of the experimental set-up and the sampling of different settling stages are described in Abramova et al., 2018 (manuscript). The data is available at the NCBI under BioProject ID PRJNA528777. Briefly, 1–2 days old cyprid larvae were left to settle in petri dishes for 4–5 days. After that time-period, we collected several settling/developmental stages, including free swimming cyprids, cyprids during close search, early attached cyprids and early metamorphosed juveniles. Each sample contained 20 individuals and was collected in three independent replicates. RNA was extracted with previously optimized protocol [27] and sent for sequencing at the Science for Life Laboratory in Stockholm. Obtained RNA-seq data was assembled with Trinity software and used to quantify transcripts’ expression levels. Subsequently, differential gene expression analysis was performed with the edgeR package using the likelihood ratio test [40]. Genes that had adjusted p-values equal or smaller than 0.05 and a fold change more than 4 were reported.

## Results

### Antennular transcriptome

In order to enrich for antennual sensory genes we dissected and collected antennule pairs from 150 cyprid individuals (Fig 1) from which we extracted RNA. Sequencing of the obtained RNA generated more than 260 million good quality read pairs (in total roughly 89 Gbp). *De novo* assembly was perfomed using Trinity [29] and clustered with CD-HIT to reduce redundancy. The resulted assembly contained 214,110 Trinity transcripts representing 142,285 Trinity genes (Table 1). Average contig length and N50 value were 417 and 458, respectively. Transdecoder was used to identify candidate coding regions within the assembled contigs resulting in total of 90,896 ORFs from which 15,931 were complete ORFs.

**Table 1:** *De novo* antennular transcriptome assembly and annotation statistics, nt = nucleotides.

Total assembled bases	89 341,753
Total Trinity transcripts*	214,110
Total Trinity genes	142,285
Average contig length (nt)	417
Contig N50 (nt)	458
ORFs**	90,896
Annotated genes	15,953

*after CD hit clustering and reducing redundancy by removing duplicates at 95% identity level.

**all types of ORFs predicted by Transdecoder (15,931 complete, 35,284 internal, and 39,654 partial)

To assess the quality and duplication level in the antennular transcriptome, we ran the BUSCO set comprised of arthropod single copy orthologs (S1 File). The analysis revealed a duplication level of 18%. Among all transcripts, 7.4% were predicted to have complete ORFs (Table 1).

### Transcriptome annotation

**Fig 2:**
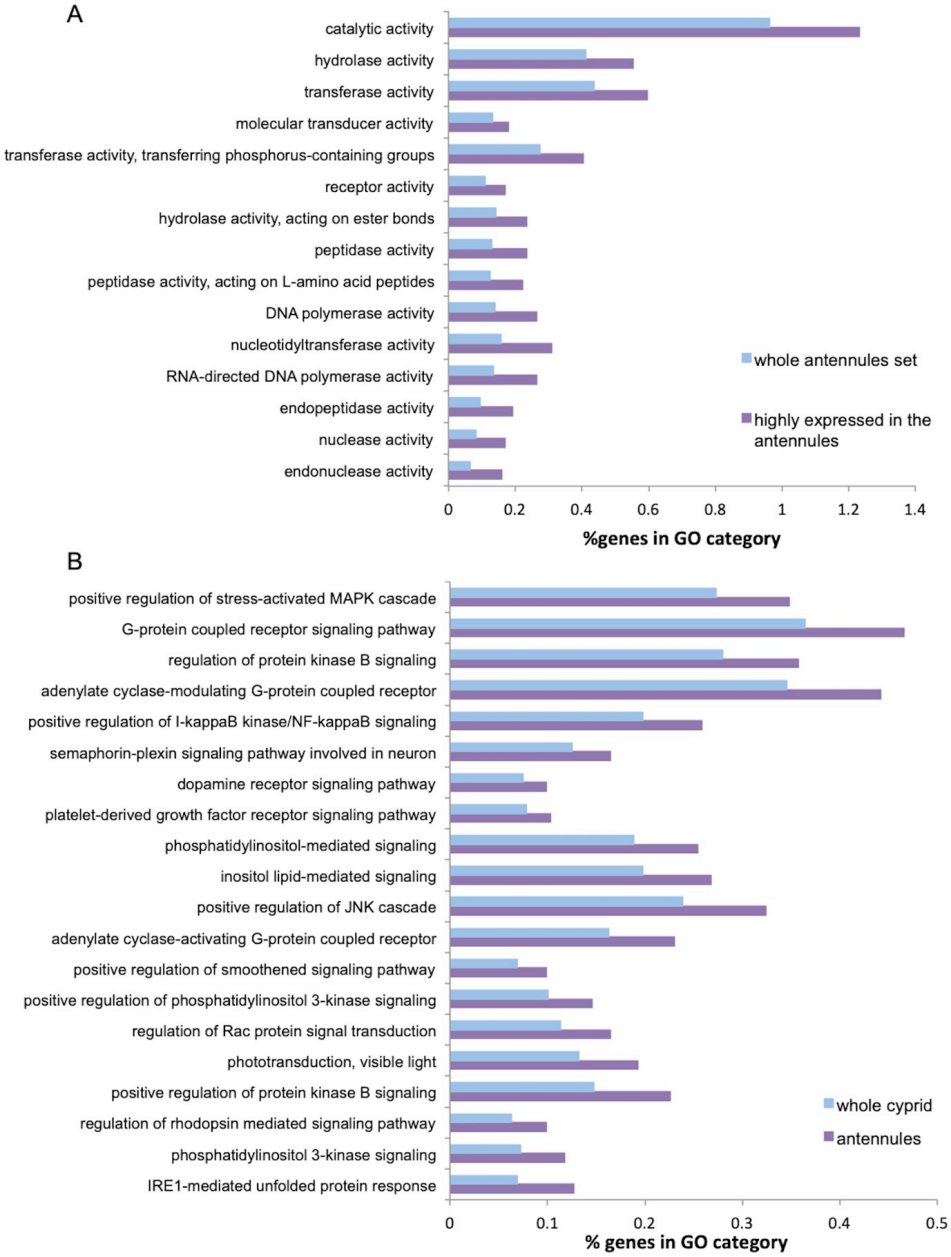
Functional characterisation of the antennular dataset. (A) GO enrichment for the top 15 Molecular Function categories of the subset of genes highly expressed in antennular dataset (at least 10 times more than in the cyprid) compared to the whole antennular dataset as a background. Each GO term has at least 15 genes assigned in the antennular dataset. (B) GO enrichment comparison of the genes in the Biological Process category ‘signal transduction’ (GO:0007165) between the antennules and whole cyprid datasets. Categories are sorted—left to right—based on the fold-change differences between the two dataset. Each GO term has at least 20 genes assigned in the antennular dataset.

We performed functional annotation of the antennular transcriptome using Trinotate, resulting in 15,953 annotated transcripts, which is similar to the number of functionally annotated contigs in the whole cyprid *de novo* transcriptome assembly (17,015) (S1 File; unbuplished data). GO enrichment analysis was done using the annotated transcripts. In the subset of genes that were at least ten times more abundant in the antennules than in the whole cyprid dataset, we observed several GO terms enriched in the Molecular Function categories related to the sensory function performed by antennules. In particular, “receptor activity”, “molecular transducer activity” and “transferase activity” were among the top 15 enriched GO terms for the genes with higher expression in the antennules than in the whole body (Fig 2A). To get an overview of the differences between genes expressed in the antennules and whole body datasets, we also mapped GO terms from both datasets to the GO slim subset. Comparison revealed that the distribution of GO terms are overall very similar between the two datasets (S1 Fig). However, when we looked at the GO terms assigned to the GO slim category “signal transduction” (GO:0007165) we could see that the antennular dataset showed enrichment for signaling related genes compared to the whole cyprid transcriptome, e.g. genes involved in “phosphatidyl 3-kinase signalling” and “adenylate cyclase-activating G-protein coupled signalling” (Fig 2B).

### HMM models and search results

In order to find sensory receptor genes that are expressed in the cyprid antennules, we constructed nine HMM models based on previously annotated sensory receptor sequences from arthropods. Our HMM searches of the antennular transcriptome identified dozens of sensory receptor candidates (S5 File). However, a majority of these candidates were partial ORFs found on short contigs, too short to be unambiguously identified as belonging to a particular class of receptors. These partial candidate sequences were extended when possible, using transcriptomic contigs from whole cyprid and adults, and these extended candidates were confirmed using DNA data. This resulted in the identification of several putative IRs, including IR25a, IR8a, and IR93a as well as four additional divergent IRs. Search for TRP receptors revealed many false positives comprising fragments of other proteins containing ankyrin repeates also present in TRPs. The filtering resulted in identitifcation of 13 TRP candidates in the antennular dataset. In addition, we found one GR-like candidate expressed in antennules and five more were retrieved from the adult transcriptome assembly. Identified GR candidates were all partial and had a very low identity to the known GRs (26–38%) (S3 File). In particular, the identified GR-like receptor sequence from antennules was 171 aa long containing only two transmembrane domains out of seven normally present in GRs. It had 30% identity to the previously reported *B*. *amphitrite* partial (80aa) GR-like sequence [12] and 38% identity to *Drosophila hydei* putative gustatory receptor 98b (XP_023161714.1). Furthermore, the identified *B*. *improvisus* GR-like receptor had the signature amino acid domain of the GR family [41], the “TYxxxxxQF” motif (S4 File).

We also searched for the ORs, odorant-binding proteins and chemosensory proteins, three groups of proteins that are important for chemosensory function in several arthropod groups. We did not detect any clear candidates for these classes, in line with earlier results indicating that these proteins are specific to Hexapoda [16].

### Ionotropic receptors

**Fig 3:**
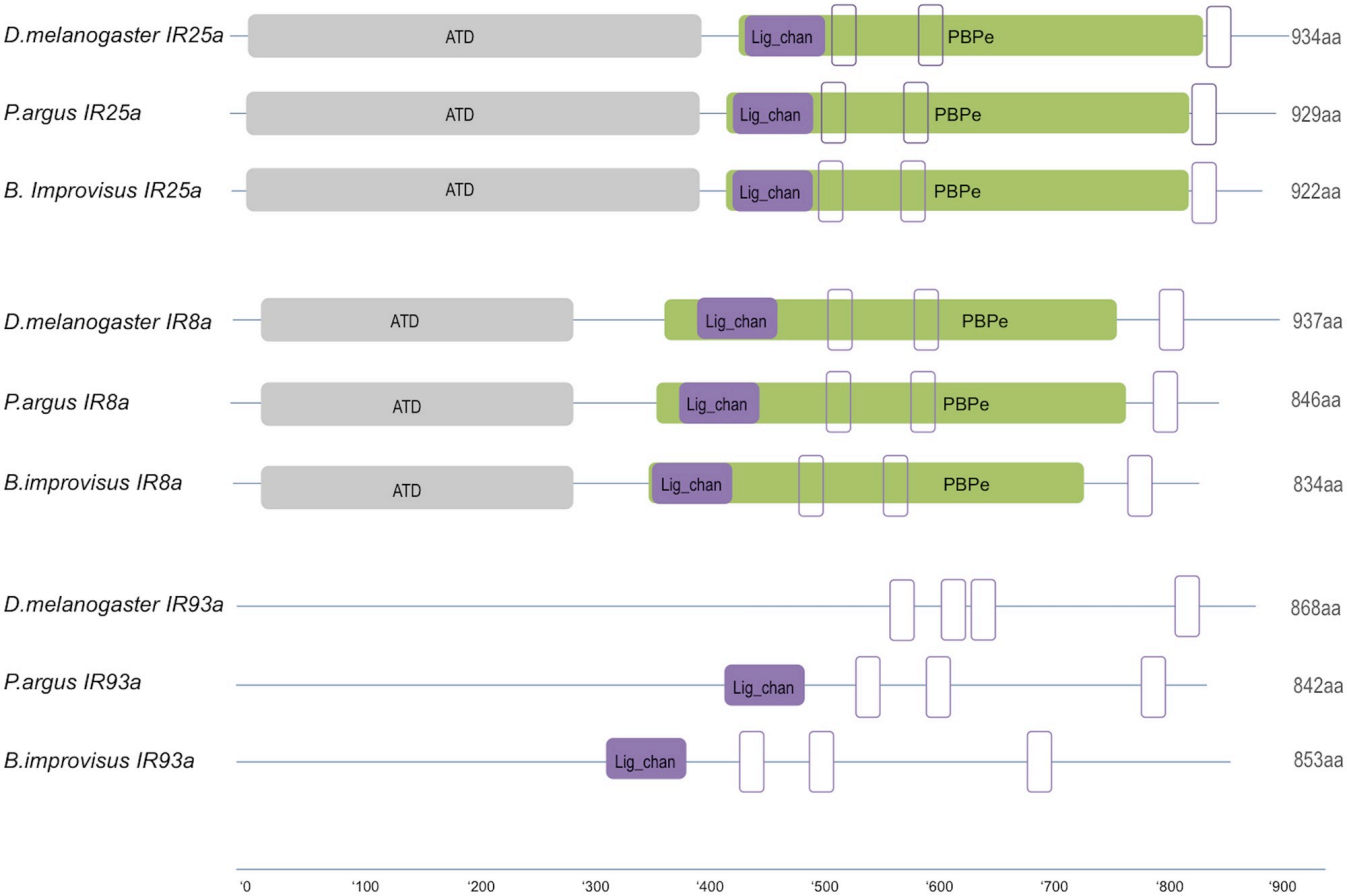
The comparison of protein domain organisation of IR25a, IR8a and IR93a from *D*. *melanogaster*, *P*. *argus* and *B*. *improvisus*. ATD amino-terminal domain (grey), Lig_chan Ligated ion channel L-glutamate- and glycine-binding site (PF10613) (violet) overlapping with PBPe (PF00060) domain (green). White boxes represent transmembrane domains predicted with TMHMM Server v. 2.0.

After filtering of the initial candidates scored by HMMs, we identified several putative IRs in our antennual RNA sequences (Table 2). One of the candidates, *Bimp*IR25a, revealed 56% identity to the spiny lobster *P*. *argus* subunit IR25a and 52% identity to the *D*. *melanogaster* IR25 at the amino acid level. Domain prediction with SMART showed the presence of the two characteristic IR domains: a conserved domain corresponding to the periplasmic substrate binding protein family (PBPe) (E-value 9.37e^-60^), overlapping with a Ligated ion channel L-glutamate- and glycine-binding site (E-value 2.46e^-19^) (Fig 3).

**Table 2:** *B*. *improvisus* candidate IRs, BLASTp search was carried against the Arthropod nr NCBI database.

Gene name	Accession numbers	Length (aa)	Best blastp hit	E-value	Identity
*Bimp*IR25a	MK093206	922	olfactory ionotropic receptor IR25a, *Panulirus argus AGJ51188*.*1*	0.0	56%
*Bimp*IR8a	MK093207	860	Ionotropic receptor 8a *Blattella germanica*, *PSN54615*.*1*	0.0	50%
*Bimp*IR93a	MK093208	849	olfactory ionotropic receptor IR93a, *Panulirus argus AGJ51190*.*1*	0.0	41%
*Bimp*IR1	-	525	ionotropic receptor 129, *Blattella germanica PSN40732*.*1*	3e-23	30%
*Bimp*IR2		542	ionotropic receptor 76a *Daphnia magna*, *KZS15214*.*1*	2e-77	35%
*Bimp*IR3	-	469	ionotropic receptor 21a-like *Eurytemora affinis*, *XP_023320534*.*1*	1e-35	26%
*Bimp*IR4	-	357	variant ionotropic glutamate receptor *Coenobita clypeatus*, *CEF34384*.*1*	3e-29	27%

Furthermore, we found one IR8a candidate in *B*. *improvisus* corresponding to a protein of 834aa. Sequence alignment revealed 49% identity to the *P*. *argus* IR8a (AGJ51189.1) and 44% to *D*. *melanogaster* IR8a (NP_727328.1). Sequence analysis showed the presence of both IR-characteristic domains in *Bimp*IR8a (Fig 3). Furthermore, one gene encoding IR93a was found in the *B*. *improvisus* antennular dataset. The sequence corresponded to 853 aa, with 40% identity to *P*. *argus* IR93a protein sequence (AGJ51190.1), and contained the ion channel pore region and a part of the ligand-binding domain.

The remaining identified IR candidates had low similarity to the known IRs and iGluRs (26–35% ID) (Table 2). Despite the fact that the overall sequence identity to the known IRs and iGluRs was low, four of the candidates contained parts of the ligand-binding domain and ion channel pore region; we named these *Bimp*IR1 to 4. We found that two of these putative IR candidates, *Bimp*IR4 and *Bimp*IR5, were located tail to tail on different strands of the same genomic scaffold of 42,766 kb (S2 Fig). It has been previously observed that IRs are often organised in tandem arrays in the genome in *Drosophila*, as a result of recent local gene duplications [16]. We found only one such example in our *B*. *improvisus* contigs likely due to the fragmented nature of the genome assembly.

**Fig 4:**
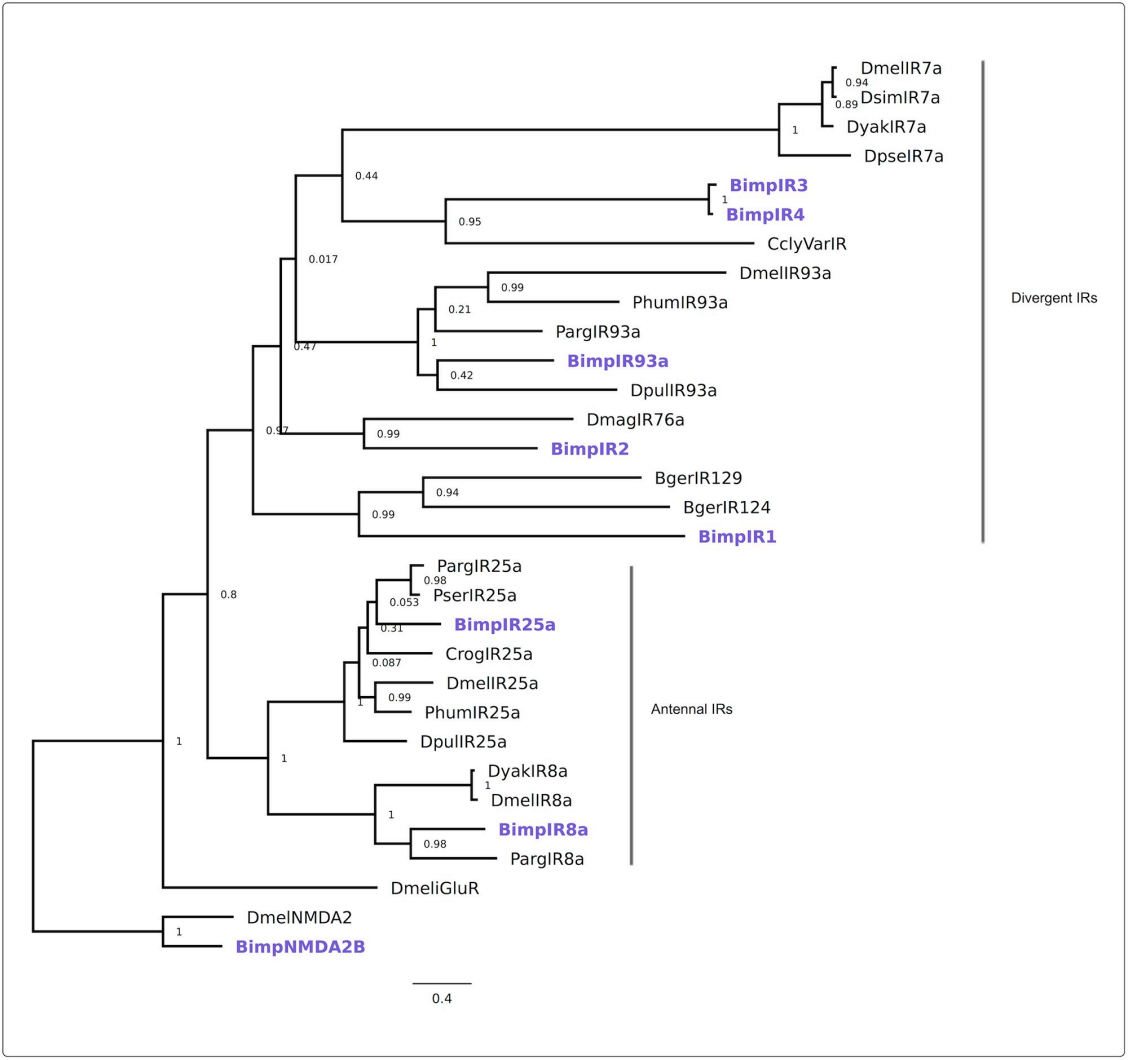
Maximum-likelihood phylogeny for *B*. *improvisus* candidate IRs including IR25a, IR8a, IR93a, and four divergent *Bimp*IR1-4. IR candidate amino acid sequences from *B*. *improvisus* were aligned with reference sequences from other arthropod species from NCBI: Parg *P*. *argus*, Dmel *D*. *melanogaster*, Phum *Pediculus humanus*, Dpul *D*. *pulex*, Ccly *Coenobita clypeatus*, Dsim *D*. *simulans*, Dyak *D*. *yakuba*, Dpse *D*. *pseudoobscura*, Dmag *D*.*magna*, Bger *B*. *germanica*, Crog *Caligus rogercresseyi*, Pser *Palaemon serratus*. See S2 File for the accession numbers. NMDA type receptors from *D*. *melanogaster* and *B*. *improvisus* were used as an outgroup. The numbers indicate bootstrap values for each branch. The scale bar indicates the inferred number of substitutions per site.

To classify the putative IRs a phylogenetic analysis was performed. The analysis showed that IRs formed two clusters in the phylogenetic tree of IRs (Fig 4). One cluster contained *Bimp*IR25a and *Bimp*IR8a together with the IR25a/IR8a and ancestral iGluRs from *Drosophila* and several crustacean species used as a reference. Another cluster comprised species-specific/divergent IRs. This cluster contained *Bimp*IR93a that formed a clade with IR93a orthologs from the reference species, and other divergent IRs showing similarity to *B*. *germanica* IR214 and *D*. *magna* IR76a.

**Fig 5:**
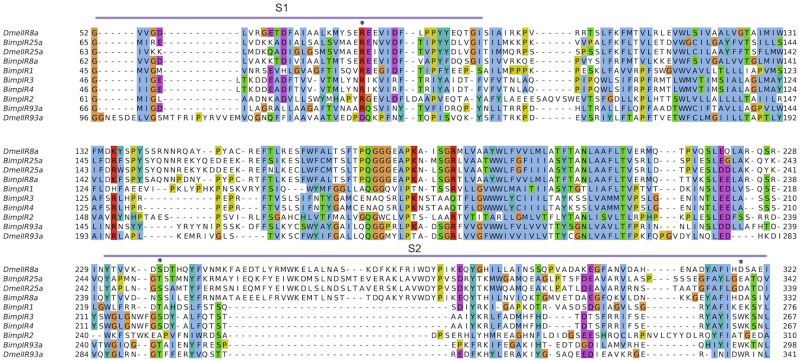
Alignment of PBPe domains from *D*. *melanogaster* IR25a, IR8a, IR93a and candidate *B*. *improvisus* IRs. The three glutamate binding positions characterised from Benton et al. (2009) are marked with asterisk. S1 and S2 stand for ligand-binding lobes of the “Venus flytrap” of the iGluRs.

We compared the ligand-binding domains of the identified antennual *B*. *improvisus* IRs with the corresponding *D*. *melanogaster* IR25a and IR8a that retain most of the three conserved glutamate-interacting residues of iGluRs (Fig 5). *B*. *improvisus* IR25a has retained arginine (R), threonine (T) and aspartate/glutamate (D/E) residues characteristic for the iGluRs, while IR8a has a threonine residue replaced by isoleucine (I) in the S2 domain. We observed that among our putative IR candidates a few, especially divergent IRs, lack one or more of these conserved residues suggesting that they are not binding glutamate like in the case of *Drosophila* IRs [15].

## Transient receptor potential channels

**Fig 6:**
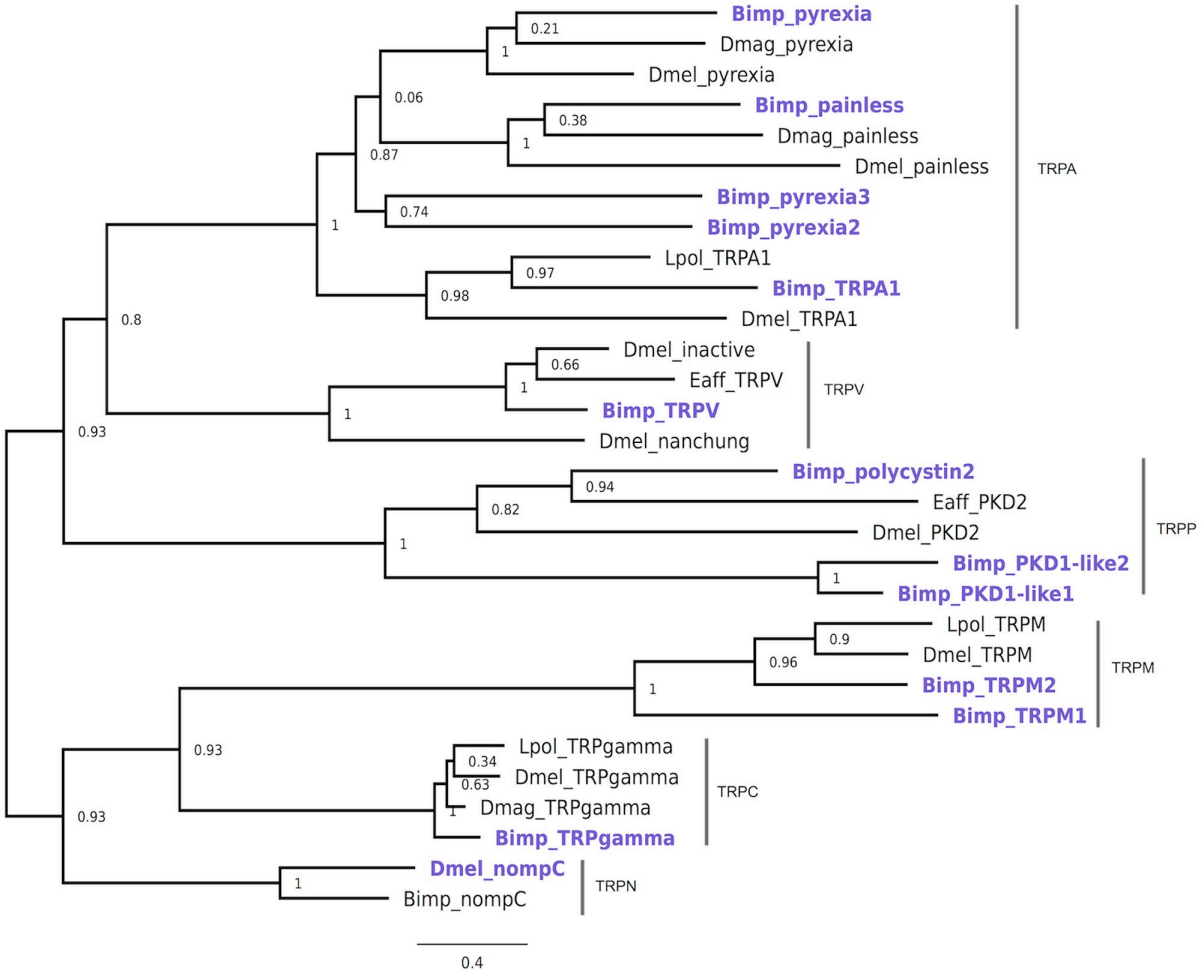
*B*. *improvisus* and other arthropod TRP channels. Amino acid sequences of candidates from *B*. *improvisus* (*Bimp*) and other arthropod species (accession numbers S2 File) were aligned using MUSCLE program following by PhyML for maximum likelihood analysis using online phylogeny.fr server. *B*. The numbers indicate bootstrap values for each branch. The scale bar indicates the inferred number of substitutions per site.

Using the HMM models when searching the *B*. *improvisus* antennular dataset we also identified 13 sequences representing putative TRP receptors (Table 3). Further, to assign the candidates to TRPs subfamilies we made maximum-likelihood phylogeny including TRPs from *Drosophila* as well as from several crustacean species (Fig 6). This analysis revealed strong evidence that five of our 13 TRP candidates belong to the TRPA family, including TRPA1, *painless* and three *pyrexia*-like sequences. In addition, we found two TRPM-like transcripts, three *polycystic kidney disease*-like proteins belonging to TRPP subfamily, and transcripts corresponding to TRPV and TRPγ.

**Table 3:** Table of TRP candidates identified from cyprid antennules transcriptome.

Gene name	Accession numbers	Length (aa)	Best blast hit	E-value	Identity
*Bimp*_TRPA1	MK093202	1262	transient receptor potential cation channel subfamily A member 1 homolog isoform X1 *Limulus polyphemus*, *XP_022241898*.*1*	0.0	47%
*Bimp*_painless	MK093200	909	transient receptor potential cation channel protein painless-like *Eurytemora affinis*, *XP_023338859*.*1*	4e-130	33%
*Bimp*_pyrexia1	MK093194	1191	transient receptor potential channel pyrexia-like *Zootermopsis nevadensis*, X*P_021941613*.*1*	2e-177	37%
*Bimp*_pyrexia2	MK093195	881	transient receptor potential channel pyrexia *Microplitis demolitor*, *XP_008553590*.*1*	2e-73	32%
*Bimp*_pyrexia3	MK093205	923	transient receptor potential channel pyrexia-like *Hyalella azteca*, *XP_018007126*.*1*	9e-107	34%
*Bimp*_nompC	MK093193	986	ion channel nompc *Culex quinquefasciatus*, *XP_001842099*.*1*	0.0	46%
*Bimp*_TRPM1	MK093204	1002	transient receptor potential cation channel trpm-like *Eurytemora affinis*, *XP_023322531*.*1*	0.0	35%
*Bimp*_TRPM2	MK093196	1654	transient receptor potential cation channel trpm *Nilaparvata lugens*, *XP_022186364*.*1*	0.0	47%
*Bimp*_TRPV	MK093197	762	TRP channel protein inactive *Nephotettix cincticeps*, *ATU07274*.*1*	0.0	63%
*Bimp*_TRPgamma	MK093198	846	transient receptor potential-gamma protein isoform X2 *Vollenhovia emeryi*, *XP_011868062*.*1*	0.0	67%
*Bimp*_PKD1-like1	MK093199	976	polycystic kidney disease protein 1-like 2 *Limulus polyphemus*, *XP_022236079*.*1*	0.0	45%
*Bimp*_polycystin2	MK093201	735	Polycystin-2-like *Limulus polyphemus*, *XP_013778837*.*1*	0.0	60%
*Bimp*_PKD1-like2	MK093203	2422	polycystic kidney disease protein 1-like protein 2-like protein *Euroglyphus maynei*, *OTF78065*.*1*	0.0	32%

In addition, one contig was annotated as the ion channel nompC (“no mechanoreceptor potential C“), the first TRP receptor described from *Drosophila*. This protein showed 49% identity (E 0.0) with *D*. *melanogaster* nompC (NP_001097089.2). *Drosophila* nompC is 1,726 aa long and contains 29 ankyrin repeats, however, the *B*. *improvisus* sequence is partial (986 aa), lacking part of the N-terminus and containing only eleven ankyrin repeats.

The phylogenetic analysis revealed that *B*. *improvisus* TRPs cluster well together with corresponding representatives of well-studied classes from *Drosophila* and other arthropods with high support (Fig 6).

### Expression of the sensory receptors during cyprid settlement

**Fig 7:**
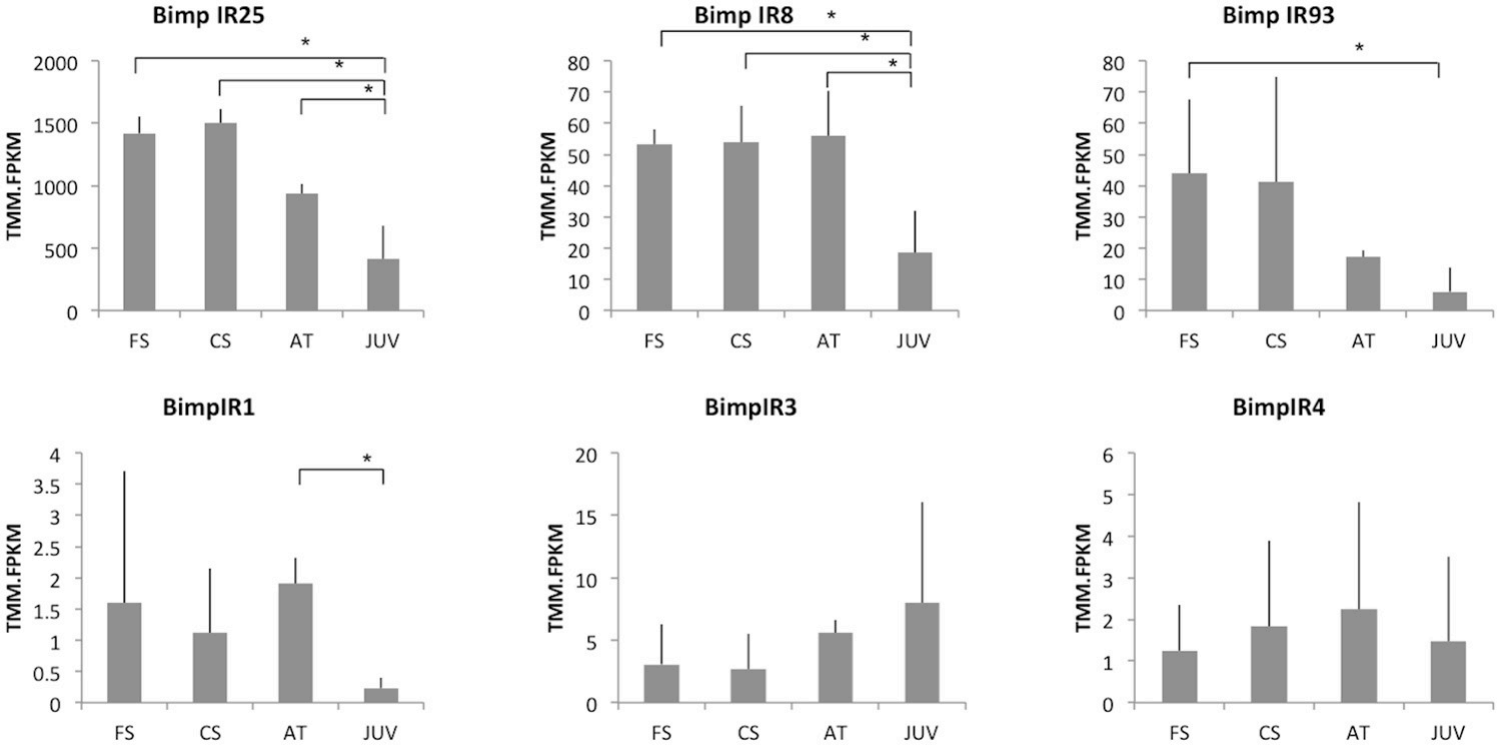
*B*. *improvisus* IRs expression during settlement. FS free-swimming, CS close search, AT early-attached stage, JUV juvenile. Significant changes in gene expression (FDR 0.05) are indicated with an asterisk.

We performed differential gene expression analysis to investigate how the identified sensory receptor candidates were expressed during distinct settlement-stages, i.e. free swimming cyprids, cyprids during close search, early attached cyprids and early metamorphosed juveniles (Fig 7 and S2 Table). Expression of the ionotropic receptors appears to be highest in the free-swimming and close-search stages and decreases substantially after attachment of cyprids, with the exception for IR8a expression that remained high in the attached stage followed by a roughly 3-fold decrease in the juvenile. The overall considerably higher expression of the IR25 candidate (roughly 1,500 FPKM in comparison to about 50 FPKM for the other two IRs) is compatible with IR25 being the common co-receptor for many of the more specific IRs [11,15], thus being more highly expressed or/and in a greater number of cells compared to the other IR classes. In contrast, the divergent IRs demonstrated comparatively low and highly variable expression, consistent with their suggested cell-specific expression [17]. The divergent IR2 as well as GR-like receptor were not found in the settling assembly, probably due to extremely low expression levels.

**Fig 8:**
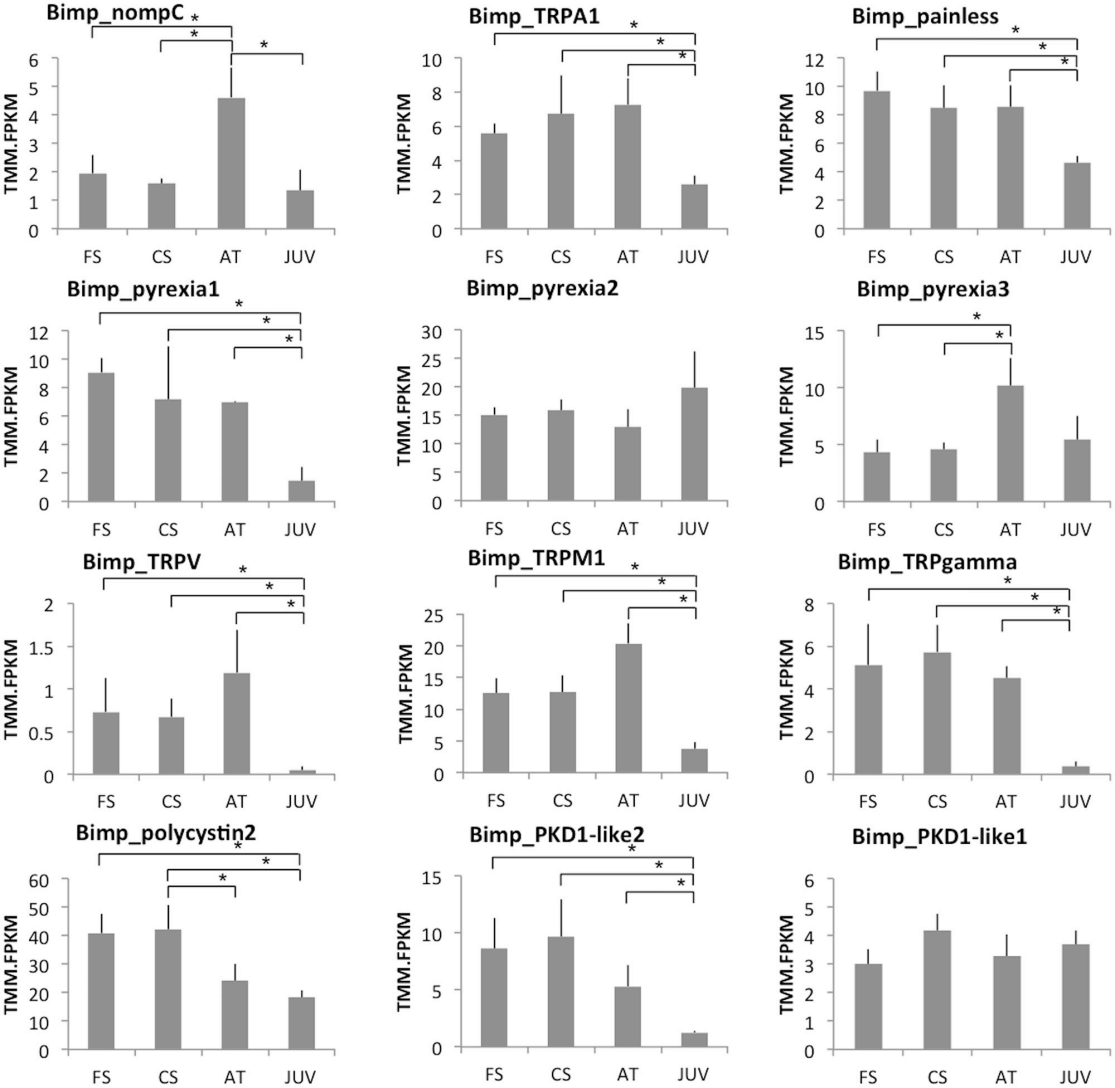
Expression of the *B*. *improvisus* TRP channels during settlement. TRPM2 shows exactly the same expression pattern as TRPM1 and was not shown. FS free-swimming, CS close search, AT early-attached stage, JUV juvenile. Asterisk shows significant change in gene expression FDR 0.05.

For the TRP channels we observe quite variable expression during settlement (Fig 8). Similar to the IRs, the majority of the candidate TRPs are downregulated in the juvenile stage compared to the cyprid stages. However, nompC showed high expression in the attached stage, whereas pyrexia 2 and PKD1-like 1 levels did not change significantly during the settlement.

## Discussion

A remarkable feature of barnacles is their complex settlement behavior allowing them to find a suitable attachment site for survival and reproduction. During settlement, barnacle larvae display a high degree of specificity towards surface chemistry and structure, as well as the ability to discriminate between conspecific and other barnacle species’ cues [42]. While some of the cues triggering settlement have been characterised, the exact molecular mechanisms of how cyprids receive these cues remain largely unknown. In the present study we identified three types of sensory receptors in the barnacle *B*. *improvisus*, olfactory ionotropic receptors, gustatory-like receptors and mechanosensory transient receptor potential channels, present in the cyprid antennules that most likely are involved in the sensing of settlement cues. We could not find any signs of odorant receptors, chemosensory or odorant-binding proteins known to have chemosensory function in other invertebrate species. These results are consistent with the reported absence of these genes from other crustaceans, i.e. the hermit crab and lobster antennal transcriptomes [11,13]. Consistent with the number of GRs found in the antennular flagellum of spiny lobster *P*. *argus* [43], we identified only one GR-like receptor in cyprid antennules, and five additional in the adult transcriptome. GRs have a diverse set of functions ranging from detection of sugars and carbon dioxide to sensing of heat and light [41]. Being one of the most ancient receptors in Arthropoda, GRs expanded in some lineages, eg *D*. *pulex* has 58 GRs [14], and were completely lost in others. Unfortunately, the fragmented nature of our datasets did not allow for more in-depth analysis and most likely underestimates the true number of GR genes in *B*. *improvisus*.

Our transcriptomic data from antennules of cyprids show the presence of several transcripts from ionotropic receptors. Evidence from recent studies suggests that IRs are widely distributed among crustaceans and are the main candidates to initiate chemosensory signaling [11]. The total number of IRs varies greatly from species to species with the hermit crab *C*. *clypeatus* having 27 and *D*. *pulex* having 85 IRs [16,18]. Based on the protein sequences and phylogenetic analysis we identified and classified the *B*. *improvisus* IR25a, IR8a and IR93 subunits, as well as four divergent IRs. Furthermore, we identified dozens of sequences in the antennal dataset, as well as in whole-cyprids and adults, that had sequence homology to IRs and iGluRs and might belong to the divergent IRs, however, the fragmented nature of the data does not allow to unambiguously classify them. The total size of the IR repertoire in *B*. *improvisus* is currently difficult to estimate without a better genome assembly as a reference.

According to the current insect model, the common IR25a and IR8a subunits are present in each olfactory receptor neuron and form heteromers with cell-specific divergent IRs rendering ligand specificity of individual sensory cells [17]. Indeed, we observed that *B*. *improvisus* IR25a showed considerably higher expression in cyprids compared to other identified IRs, while the expression level of the divergent IRs were considerably lower consistent with their cell-specific role [11]. Our data show that common IR subunits (IR25a, IR8a and IR93a) in *B*. *improvisus* were highly expressed during surface exploration and early attachment, suggesting possible importance in detecting settlement cues. Noticeably, these IR subunits are also highly expressed in the settlement stage of the parasitic salmon louse *Lepeophtheirus salmonis* [44] and crucial for the identification of the host species [44]. Furthermore, members of the IR20 clade in *Drosophila* are suggested to be involved in sensing pheromones and regulation of courtship behaviour [45]. It is thus likely that the IRs present in the cyprids antennules could be also involved in sensing barnacle pheromones and therefore are potential targets to study pheromone-evoked settlement behavior of cyprids.

Apart from chemical cues, cyprids are thought to percieve water flow and distinguish surface structures by means of mechanosensory setae [7–9,46]. Cyprids of *B*. *improvisus* prefer to settle on smooth rather than structured surfaces [47]. According to current knowledge, mechanoreception in arthropoda is accomplished through mechanosensitive ion channels that upon stress allow the influx of cations resulting in a receptor potential [19]. Based on the effect of specific agonists of calcium channels, it has been suggested that TRP channels mediate calcium entry in neurons when the cyprid senses a favourable substratum [22]. With HMM models based on the known arthropod TRP receptors, we detected 13 TRP candidates in the antennular transcriptome of *B*. *improvisus* cyprids (Table 3). This number corresponds roughly to the number of TRP genes found to be expressed in *D*. *pulex* transcriptome, with the exception that TRPP genes seems to be absent in *Daphnia* [48].

We identified five candidates belonging to TRPA receptors including TRPA1, *painless* and three *pyrexia* genes [20]. TRPA1 is involved in sensing of volatile molecules in *Drosophila* and nose-touch responses and foraging in *Caenorhabditis elegans* [49,50]. The *B*. *improvisus* homolg of TRPA1 was highly expressed during exploratory stages and early attachment. The *painless* gene in *Drosophila* larvae is involved in behavioral responses to thermal and mechanical stimuli as well as playing a role in reception of pheromones and sexual behavior in adults [51–53]. Interestingly, benzyl isothiocyanate that is an activator for both TRPA1 and *painless* channels had an effect on the settling of cyprids, inhibiting settlement at lower concentrations and stimulating at higher, suggesting that TRP channels activation generate Ca^2+^ signals that coordinate the settlement process [22]. Like in *D*. *pulex* [20], the *pyrexia* gene appears to be expanded in *B*. *improvisus* being represented by three paralogues. While the role of *pyrexia* in crustaceans remains to be established, this gene detects a range of temperatures and negative geotaxis in *Drosophila* and contributes to host-seeking in *Anopheles gambiae* [54].

Furthermore, we found two TRPM-like transcripts among *B*. *improvisus* TRP candidates. In *Drosophila*, TRPM is mainly involved in ion homeostasis during larval development [20,49], whereas in mammals TRPM is expressed specifically in the olfactory epithelium and is involved in transducing pheromone signals [55]. The fourth segment of the cyprid antennule carries an aesthetascs-like setae that was suggested to sense waterborne chemical cues [7]. In this perspective, future studies on the localization of the expression of particular types of TRPs by, for example, *in situ* hybridization are of a great interest. Two of the other identified TRP channels, namely TRPgamma and TRPV, showed expression patterns similar to the TRPM with high expression in the exploratory stages and decreased expression in the juvenile stage. TRPgamma is localised in the proprioceptive cells and contribute to fine motor control in *Drosophila* and *C*. *elegans* [56]. Fine motor control in flies is required for challenging tasks, which rely on coordinating a repertoire of fine movements, including subtle changes in body angles and leg positions [56], which resemble the complex movements of cyprid antennules during the surface exploration [7]. The TRPV receptor is considered as a molecular integrator of noxious stimuli ranging from pungent natural products, such as capsaicin, to acidic environment and high temperatures [57]. A recent study suggested that cyprid settlement could be akin to the behavioral responses of *Drosophila* to noxious stimuli mediated by TRPs [22]. In particular, surface avoidance would be mediated by noxious stimuli-sensing channels, cancelling the signals that complete settlement. Therefore, further investigation of the antennual TRPs could bring new insights into the cyprid decision-making process.

The current study provides the first molecular evidence of the existence of IRs and TRP channels in barnacles cyprid antennules and their variable expression during the settling, thus suggesting possible roles for them in the process of barnacle settlement. The generated datasets bring new opportunities to further investigate settling behavior and provide possible new targets for development of antifouling agents with more specific effects on barnacles.

## Supporting information

S1 File Annotation and BUSCO assessment of the datasets. (XLSX)

S2 File Arthropod sequences used as a reference for the *B*. *improvisus* IRs phylogenetic analysis. (XLSX)

S3 File GR-like candidates identified from antennular and adult *B*. *improvisus* transcriptomes. (PDF)

S4 File *B*. *improvisus* GR-like fragment alignment. (PDF)

S5 File Results of the searches with constructed HMM models. (PDF)

S6 File Fasta file with amino acid sequences described in the text. (TXT)

S1 Fig Comparison of the distribution of GO slim categories between whole body cyprid and antennular dataset. (PNG)

S2 Fig Location of the putative IR candidates on the genomic scaffold. (PNG)

S1 Table The number and taxonomic representation of sequences used to construct HMM models. (XLSX)

S2 Table EdgeR results for the receptor candidates. (XLSX)
