# Supplementary material for: Sensory receptor repertoire in cyprid antennules of the barnacle Balanus improvisus
Source: PLoS One. 2019 May 2;14(5):e0216294. doi: 10.1371/journal.pone.0216294 (PMC6497305; doi:10.1371/journal.pone.0216294)
Supplement: S3 File — (PDF) [file pone.0216294.s003.pdf]

GR-like candidates identified from antennular and adult *B. improvisus* transcriptomes

Gene name	Transcriptome assembly	Length (aa)	Best blastp hit	E-value	Identity
Bimp_Gr1	Antennules	171	putative gustatory receptor 98b [Drosophila hydei] XP_023161714	1,00E-04	38%
Bimp_Gr2	Adult	114	gustatory and odorant receptor 24-like isoform X1 [Sipha flava] XP_025419973.1	0,002	29%
Bimp_Gr3	Adult	254	PREDICTED: odorant receptor 4 [Stomoxys calcitrans] XP_013116457.1	2,00E-05	31%
Bimp_Gr4	Adult	201	gustatory receptor 22.2 [Subpsaltria yangi] AXY87931.1	3,00E-05	31%
Bimp_Gr5	Adult	488	PREDICTED: gustatory and pheromone receptor 39a isoform X4 [Drosophila biarmipes] XP_016963786.1	0,001	31%
Bimp_Gr6	Adult	117	putative gustatory receptor 28b [Ceratina calcarata] XP_017880710.1	3,00E-04	30%
