# Supplementary material for: Sensory receptor repertoire in cyprid antennules of the barnacle Balanus improvisus
Source: PLoS One. 2019 May 2;14(5):e0216294. doi: 10.1371/journal.pone.0216294 (PMC6497305; doi:10.1371/journal.pone.0216294)
Supplement: S4 File — (PDF) [file pone.0216294.s004.pdf]

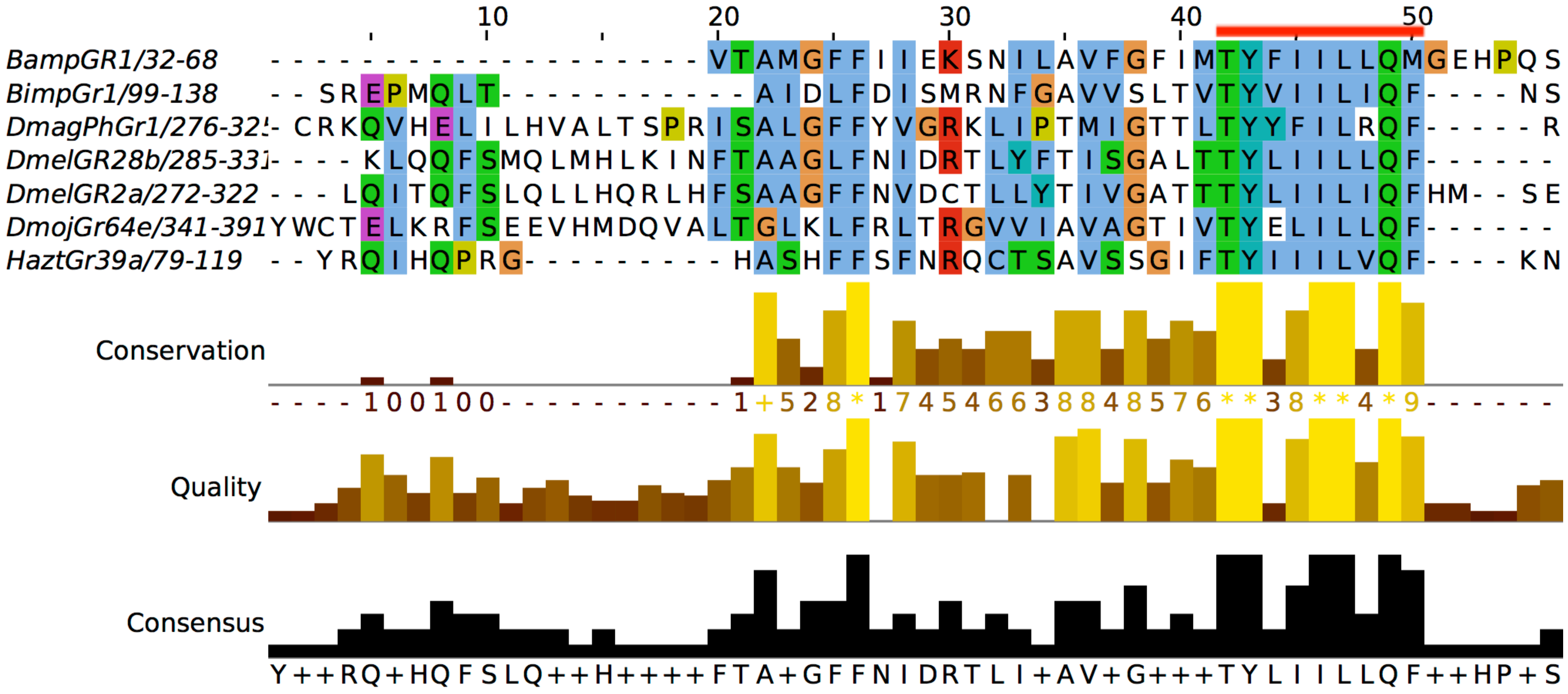

B. improvisus **GR-like fragment alignment. The multiple sequence alignment of GRs from several arthropod species (Bimp *B. improvisus*, Bamp *B. amphitrite*, Dmag *Daphnia magna*, Dmel *D. melanogaster*, Dmoj *Drosophila mojavensis*, Hazt *Hyalella azteca*) showing conserved motif "TYxxxxxQF" (underlined). The default Clustal X color scheme in the Jalview program was used.**
