# Supplementary material for: Sensory receptor repertoire in cyprid antennules of the barnacle Balanus improvisus
Source: PLoS One. 2019 May 2;14(5):e0216294. doi: 10.1371/journal.pone.0216294 (PMC6497305; doi:10.1371/journal.pone.0216294)
Supplement: S5 File — (PDF) [file pone.0216294.s005.pdf]

Results of the searches with constructed HMM models for different classes of sensory receptors in the antennular, whole cyprid and adult transcriptome datasets from *B. improvisus*. Filtering was in short based on lowest E-values and confirmation via annotated hits in the NCBI nr database - the full filtering procedure is described in the Materials and methods.

HMM model	Antennular transcriptome		Whole cyprid transcriptome		Adult transcriptome		Genome*
	Initial candidates	Candidates after filtering	Initial candidates	Candidates after filtering	Initial candidates	Candidates after filtering	
GRs	2	1	3	1	17	5	✓
ORs	0	0	2	0	1	0	
IR7a	11	0	21	0	54	0	
IR8a	4	1	5	1	53	1	✓
IR25a	50	1	47	1	48	1	✓
IR93a	14	1	22	1	45	1	✓
TRP	164	13	83	20	544	28	✓

*Genome: identified candidates were confirmed by genomic dna.
