# Supplementary figures and images for: Sensory receptor repertoire in cyprid antennules of the barnacle Balanus improvisus
Source: PLoS One. 2019 May 2;14(5):e0216294. doi: 10.1371/journal.pone.0216294 (PMC6497305; doi:10.1371/journal.pone.0216294)

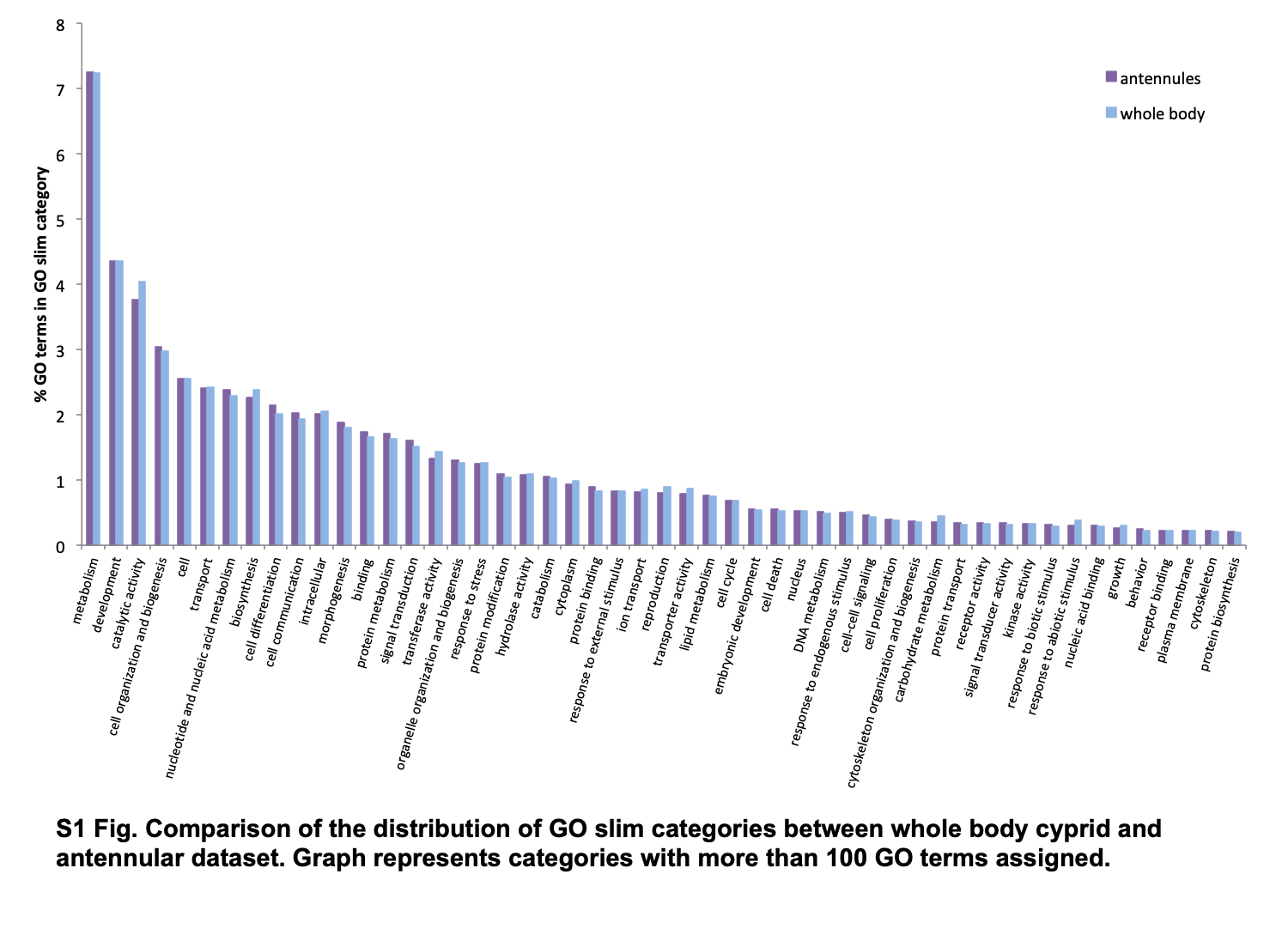

Supplement: S1 Fig — (PNG) [file pone.0216294.s007.png]

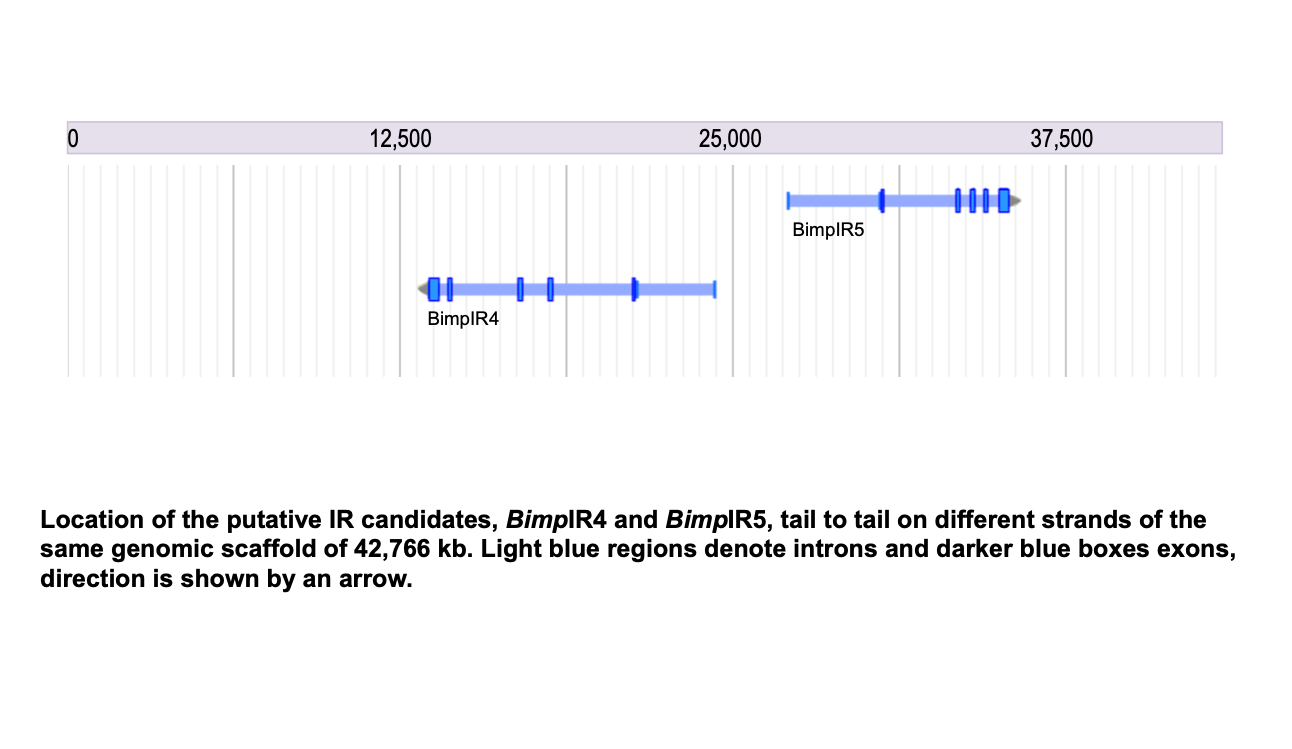

Supplement: S2 Fig — (PNG) [file pone.0216294.s008.png]
